# Correction to “Placental Mesenchymal Dysplasia: How to Approach the Diagnosis as Early as the First Trimester? A Case Report and a Review”

**DOI:** 10.1002/ccr3.73098

**Published:** 2026-07-06

S. Margheret, T. P. Sara, T. Aude, B. Frédéric, C. Marie, and G. Kalina, “Placental Mesenchymal Dysplasia: How to Approach the Diagnosis as Early as the First Trimester? A Case Report and a Review,” *Clinical Case Reports* 13, no. 10 (2025): e71194, https://doi.org/10.1002/ccr3.71194.

The first and last names of the authors were inadvertently swapped and have been corrected.

The correct author list is:

Margheret Smalaj, Sara Törnblom Paulander, Aude Tessier, Frédéric Buxant, Marie Cassart, Kalina Gajewska.

The online version of this article has been corrected accordingly.

We apologize for this error.

